# Color Variability of European Oak Veneer Packages: An Exploratory Colorimetric, Chemical, and X-Ray Diffraction Study

**DOI:** 10.3390/molecules31152712

**Published:** 2026-08-04

**Authors:** Edmund Smolarek, Kinga Szentner, Jarosław Jakubowicz, Maciej Jarzębski, Marek Wieruszewski

**Affiliations:** 1 Balti Spoon OÜ, Kupu Küla, 74610 Kuusalu Vald, Harju Maakond, Estonia; edmund@baltispoon.ee; 2 Department Mechanical Wood Technology, Faculty of Forestry and Wood Technology, Poznan University of Life Sciences, Wojska Polskiego 28, 60-637 Poznan, Poland; 3 Department of Chemistry, Faculty of Forestry and Wood Technology, Poznan University of Life Sciences, 60-637 Poznan, Poland; kinga.szentner@up.poznan.pl; 4 Institute of Materials Science and Engineering, Poznan University of Technology, Jana Pawla II 24, 61-138 Poznan, Poland; jaroslaw.jakubowicz@put.poznan.pl; 5 Department of Physics and Biophysics, Faculty of Food Science and Nutrition, Poznań University of Life Sciences, Wojska Polskiego 38/42, 60-637 Poznan, Poland; maciej.jarzebski@up.poznan.pl

**Keywords:** European oak, decorative veneer, color variability, CIELAB, extractives, lignin, cellulose crystallinity

## Abstract

European oak (*Quercus robur* L. and *Quercus petraea* (Matt.) Liebl.) is widely used for decorative veneers, but natural color heterogeneity complicates package matching and industrial sorting. This exploratory study characterized one processed veneer package from each of eight commercial provenance labels: Szczecin, Krotoszyn, Zielona Góra, Wrocław, Katowice, Krosno, and Lublin (Poland), and Wittenberg (Germany). Each package was assessed visually and by ten CIELAB measurement points, while package-level subsamples were analyzed for ethanol-soluble extractives, cellulose, lignin, and X-ray diffraction characteristics. Package means differed most strongly in lightness (L* = 59.4–69.7); however, within-package L* ranges reached 10.7 units and overlapped the differences among package means, showing that package means alone are insufficient to demonstrate provenance-based sorting. Exploratory correlations based on eight packages indicated that higher extractive content was associated with lower lightness (Pearson r = −0.80, *p* = 0.017; Spearman ρ = −0.75, *p* = 0.033), and the association remained negative in all leave-one-package-out recalculations (r = −0.96 to −0.72). In contrast, apparent Pearson lignin/color associations were weak in rank analysis and changed sign after exclusion of P5. Associations of the Segal crystallinity index with color were directional but not statistically conclusive at n = 8. All diffractograms showed cellulose I reflections near 15–17° and 22–23° 2θ; approximate CrI values ranged from 48.0% to 66.2%. The results support combined colorimetric and chemical characterization for package-level quality control, but independent replication across multiple packages per region is required before any geographic origin classifier can be validated.

## 1. Introduction

Wood is a hierarchical lignocellulosic composite in which cellulose microfibrils are embedded in a matrix of hemicelluloses and lignin, while low-molecular-weight extractives contribute strongly to optical appearance [1,2,3]. For decorative products, color, gloss, and figure are immediately visible quality attributes; among these, color directly affects commercial grading, matching, and the value of veneer surfaces [4,5,6,7,8].

Natural wood color is controlled by interacting genetic, anatomical, and environmental factors, including species, tree age, heartwood formation, site water conditions, and the concentration and distribution of extractives [9,10,11,12,13]. It is also modified by boiling, steaming, drying, heat treatment, chemical exposure, and light [4,5,14,15,16,17,18,19,20,21]. Consequently, a color difference observed after industrial processing cannot be assigned to geographic origin alone unless independent trees and production batches are replicated. A recent review summarized European oak habitats in Poland and general issues concerning oak wood color [22,23,24,25,26,27].

In European oak, *Quercus robur* L. and *Quercus petraea* (Matt.) Liebl., heartwood contains ellagitannins and other phenolic extractives that can darken the surface and alter red and yellow coordinates [13,17,28,29]. Lignin also contains chromophoric structures that contribute to visible-light absorption, whereas cellulose is comparatively colorless [30,31,32,33,34,35,36]. These molecular constituents provide a mechanistic basis for combining colorimetry with wet-chemical analysis.

The supramolecular organization of cellulose may provide an additional structural descriptor. X-ray diffraction identifies cellulose I reflections and permits an empirical comparison of the relative ordering of cellulose domains. However, crystallinity indices depend on sample preparation, preferred orientation, background treatment, and the calculation method; they must therefore be interpreted as comparative descriptors rather than absolute phase fractions.

Objective CIELAB measurement can reduce reliance on visual grading, but oak anatomy presents a practical challenge: earlywood pores, rays, annual-ring boundaries, and radial or tangential grain patterns can cause substantial variation within a single veneer package. For industrial sorting, within-package variability must therefore be evaluated together with differences among package means, rather than assuming that each measurement point is an independent replicate of regional provenance.

The present material comprised eight commercial veneer packages, each carrying a different regional provenance label. Because only one package was available for each label, region, tree/stand, harvest batch, and processing batch are confounded. This study is therefore framed as an exploratory package-level characterization and not as a replicated test of geographic origin effects.

The objectives were to (i) quantify within- and between-package variation in CIELAB coordinates, (ii) describe package-level chemical composition and cellulose I diffraction characteristics, (iii) test exploratory associations between chemical or XRD descriptors and color, and (iv) assess the extent to which the available data can support industrial package matching while identifying the replication required for future origin classification models.

## 2. Materials and Methods

### 2.1. Material

The study material consisted of eight defect-free commercial veneer packages prepared from European oak (*Quercus robur* L. and/or *Quercus petraea* (Matt.) Liebl.). Seven packages were labelled by Polish supply regions and one by Wittenberg, Germany (Table 1). All source logs were classified as Q-A, including sapwood width ≤ 3 cm, in accordance with EN 1316-1 [30]. Exactly one package represented each regional label; accordingly, the labels are used as commercial provenance metadata and not as statistically replicated geographic treatments. The seven Polish labels span western, southern, and eastern supply areas, while Wittenberg provides a neighboring German comparison. The available documentation does not establish a probability-sampling design or provide exact forest coordinates, and the material should not be interpreted as representing the full geographic ranges of either oak species.

### 2.2. Methods

Before slicing, the flitches were hydrothermally conditioned in water at 85 ± 5 °C for 20 h. The conditioned flitches were sliced to a nominal veneer thickness of 0.55 mm. The same veneer sheets then passed sequentially through three temperature zones (100, 120, and 132 °C) in a Raute GmbH industrial dryer (Raute Corporation, Nastola, Finland); the total residence time was approximately 2 min [24]. The archived record reports a final moisture content of approximately 12.6% but does not distinguish the process target from the measured final value. Subsamples used for visual, colorimetric, chemical, and XRD characterization were taken from the same production package, but the analyses were not necessarily performed on the identical physical strip.

#### 2.2.1. Visual Assessment

Visual assessment was used as a qualitative screening step, not as a quantitative color method. Veneers were inspected for knots, cracks, discoloration, mechanical damage, local fiber deformation, annual-ring regularity, ray expression, and dominant grain pattern. Straight-grained and cathedral/arched patterns were recorded because radial and tangential surface anatomy can influence the area integrated by a colorimeter aperture.

Only defect-free areas were selected for instrumental measurements. This restriction reduced obvious defect effects but did not remove natural within-package heterogeneity caused by earlywood pores, latewood bands, rays, and variation in fiber orientation.

The package photographs were acquired at a fixed distance under diffuse illumination and were used only to document surface figure. The camera model, ISO setting, exposure time, aperture, and color reference metadata were not retained in the archived dataset. The photographs are therefore illustrative and were not used to calculate color values.

The visual screening did not assign numerical grades. Its purpose was to describe dominant surface structure and to identify measurement areas free of macroscopic defects before colorimetric and compositional analyses.

#### 2.2.2. Colorimetric Analysis

Ten spatially distributed color measurements were obtained from internal veneer sheets in each package (80 measurement points in total). The ten points were subsamples for characterizing within-package heterogeneity; they were not treated as ten independent replicates of regional origin. Five-millimeter sections from the corresponding veneer blocks were imaged separately for structural documentation.

Color was measured with a PCE-XXM 30 colorimeter (PCE Instruments, PCE Deutschland GmbH-Meschede, Germany). According to the manufacturer’s specifications, the instrument uses an 8 mm measuring aperture and an LED spectral range of 400–700 nm. The instrument is supplied with a white reference; however, the archived measurement export did not document the calibration sequence or preserve the selected illuminant, observer angle, measurement geometry, or black-calibration setting. Therefore, the data permit internally consistent package comparisons but do not constitute a fully reproducible implementation of all reporting requirements of ISO 7724-2 [31]. This limitation is explicitly considered in interpreting the results.

Color was expressed in the CIELAB system as lightness (L*), red/green coordinate (a*), and yellow/blue coordinate (b*). Pairwise package mean color difference was calculated using the CIE76 expression

ΔE*ab = [(ΔL*)^2^ + (Δa*)^2^ + (Δb*)^2^]^1^/^2^

Because each package was represented by a single commercial batch, ΔE*ab values are descriptive distances between package means and not tests of geographic provenance.

#### 2.2.3. Chemical Composition Analysis

The dataset contains one reported package-level value for each chemical component in each veneer package (n = 8 package-level observations). The 0.1–0.4 mm fraction was used for wet-chemical characterization. The source records did not specify the number of laboratory replicate determinations or their variance; consequently, the chemical values are interpreted as exploratory package descriptors.

Ethanol-soluble extractives were determined gravimetrically after Soxhlet extraction, followed by drying to constant mass at 102 ± 3 °C. The procedure was an ethanol-only operational method and should not be interpreted as full conformance to TAPPI T 204, which specifies alternative solvent systems.

Cellulose was isolated by the Seifert acetylacetone/dioxane/hydrochloric acid method. The laboratory procedure used acetylacetone, 1,4-dioxane, and hydrochloric acid under heated conditions, followed by filtration, washing, drying, and gravimetric determination.

Acid-insoluble (Klason) lignin was determined with 72% sulfuric acid according to the principle of TAPPI T 222 [37,38,39,40]. All chemical contents are reported as percentages of oven dry mass.

#### 2.2.4. XRD Diffraction Analysis

One XRD specimen was analyzed from each package (P1–P8). Measurements were performed on an EMPYREAN diffractometer (Cu Kα radiation; 45 kV; 40 mA) over 5.01–110.01° 2θ with a step size of 0.0334225°. Each scan contained 10,498 angle intensity points. Main-text plots are limited to 5–50° 2θ, which contains the principal cellulose I reflections. Detailed instrumental settings and complete reference card metadata are provided in Tables S1 and S2.

An approximate crystallinity index (CrI) was calculated by the Segal expression

CrI = [I(002) − Iam]/I(002) × 100

using the maximum at 21–24° 2θ and the minimum at 17–19° 2θ [38]. Because no full background deconvolution or orientation correction was applied, CrI is used only for within-dataset comparison [39].

#### 2.2.5. Statistical Analysis

For each package, L*, a*, and b* were summarized by the mean, median, minimum, maximum, standard deviation, and range. No ANOVA or Kruskal–Wallis inference was used to attribute differences to regional origin because there was only one independent package per regional label; treating the ten within-package points as origin replicates would constitute pseudoreplication. Pearson correlations were recalculated at the package level (n = 8) using package mean color values and single package-level chemical or XRD descriptors. Spearman rank correlations and leave-one-package-out Pearson sensitivity analyses were additionally calculated for the chemical/color associations reported in Table 1. Two-sided *p*-values are reported, but these analyses are exploratory and were not adjusted for multiple testing. Calculations were performed in Statistica 13 and independently checked from the tabulated package-level data.

## 3. Results and Discussion

### 3.1. Visual Assessment

**Figure 1:**
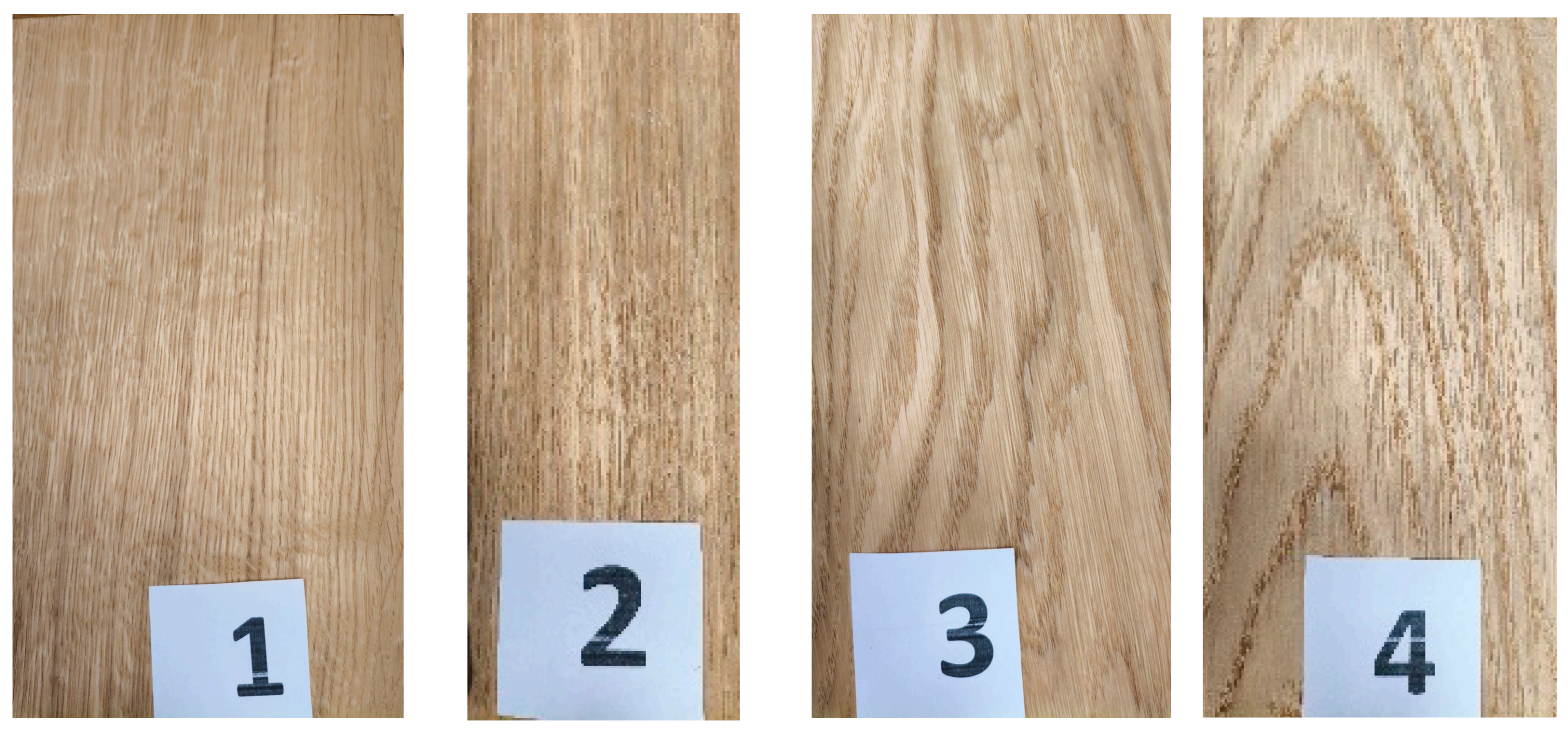

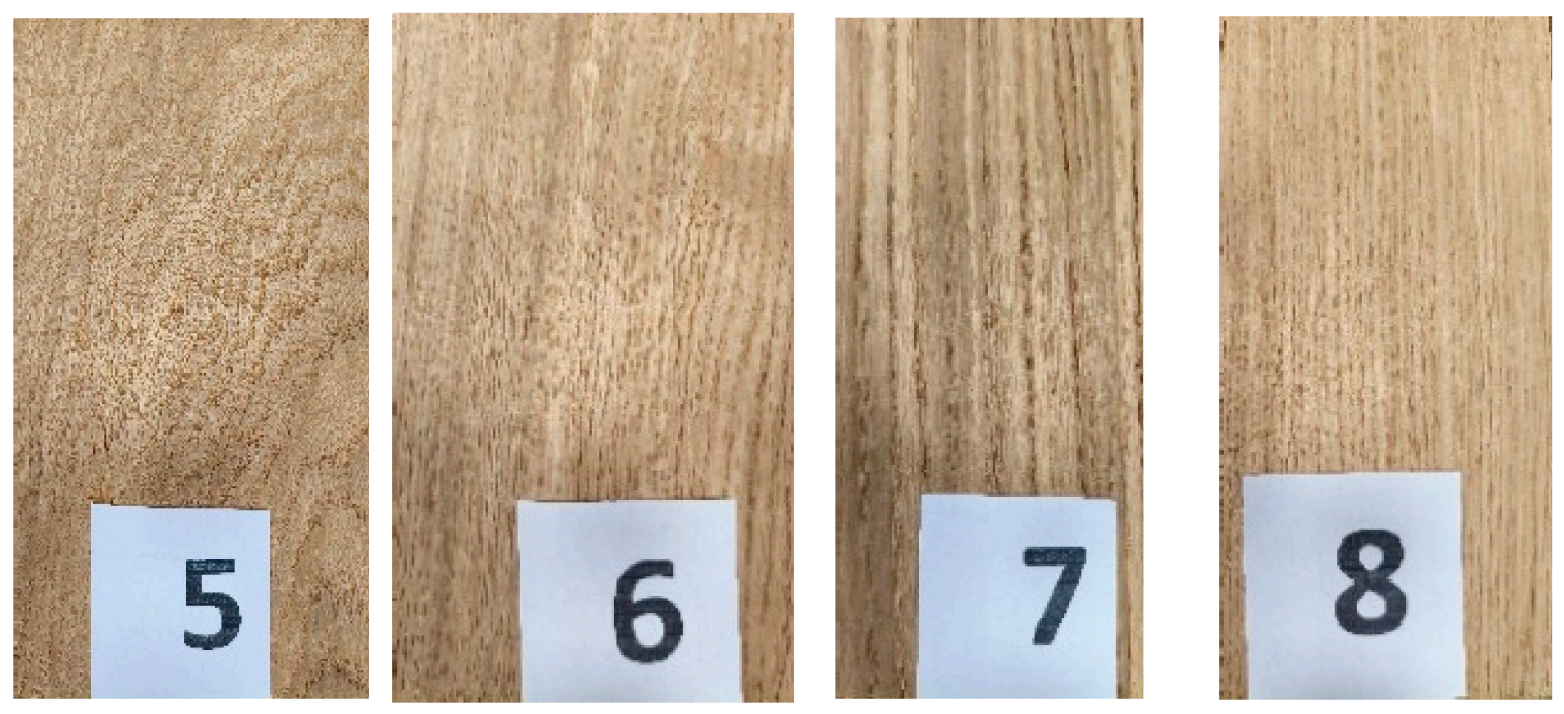
Representative oak veneer surfaces: 1 = P1 (Szczecin, Poland); 2 = P2 (Krotoszyn, Poland); 3 = P3 (Zielona Góra, Poland); 4 = P4 (Wrocław, Poland); 5 = P5 (Katowice, Poland); 6 = P6 (Krosno, Poland); 7 = P7 (Lublin, Poland); 8 = P8 (Wittenberg, Germany).

The packages shared the absence of major macroscopic defects but differed visibly in grain geometry (Figure 1). P1 (Szczecin), P2 (Krotoszyn), and P3 (Zielona Góra) were predominantly straight-grained; P4 (Wrocław) and P5 (Katowice) showed the clearest cathedral/arched figure; and P6 (Krosno), P7 (Lublin), and P8 (Wittenberg) were predominantly linear-banded. These anatomical differences are relevant because an 8 mm aperture can integrate different proportions of pores, rays, earlywood, and latewood.

The photographs support qualitative comparison of surface figure only. They do not establish color equivalence, because image exposure and color reference metadata were unavailable and instrumental CIELAB measurements were performed separately.

### 3.2. Colorimetric Analysis

Package means ranged from 59.4 to 69.7 for L*, from 6.8 to 8.7 for a*, and from 18.1 to 20.6 for b* (Table 2). Lightness therefore showed the largest separation among package means (10.3 units), followed by b* (2.5 units) and a* (1.9 units). These are descriptive package differences and must not be interpreted as replicated regional effects.

Recalculation from the package means corrected the signs and magnitudes previously reported for inter-coordinate correlations: L*-a*, r = −0.43 (*p* = 0.283); L*-b*, r = −0.34 (*p* = 0.407); and a*-b*, r = 0.40 (*p* = 0.324). None was statistically conclusive at n = 8. The earlier positive coefficients for L*-a* and L*-b* were erroneous and have been removed.

Within-package heterogeneity was substantial. The L* range varied from 1.8 units in P8 (Wittenberg) to 10.7 units in P4 (Wrocław), with a mean within-package range of 6.8 units. Thus, the variation observed inside several packages was comparable with differences among package means. This finding limits the use of a single package mean as an industrial sorting threshold.

The overall package mean values (L* = 64.7, a* = 7.5, b* = 19.4) were broadly consistent with published values for processed oak veneers, although b* was lower than the value reported by Resch et al. [21]. Differences among studies may reflect species composition, anatomy, drying conditions, extractive content, and colorimeter settings, rather than geography alone.

Because the experimental unit for provenance is the package and only one package was available per label, the distributions of the ten measurement points were not subjected to an origin-level significance test. Statements of bimodality and origin-dependent stratification have therefore been removed.

P1 (Szczecin) had the highest mean L* (69.7), whereas P5 (Katowice) had the lowest (59.4) and the highest mean a* (8.7). P4 (Wrocław) had the lowest mean b* (18.1), and P5 had the highest (20.6). These contrasts identify package profiles that may be useful for matching the studied production lots, but they do not identify regional population means.

The package mean ΔE*ab matrix (Table 3) was recalculated from the displayed L*, a*, and b* means. The smallest distances occurred between P6 (Krosno) and P8 (Wittenberg) (ΔE*ab = 1.09) and between P2 (Krotoszyn) and P6 (Krosno) (1.35); P3 (Zielona Góra) and P7 (Lublin) were also similar (1.47). The largest distance was between P1 (Szczecin) and P5 (Katowice) (10.50).

These mean distances should be interpreted together with Table 2. For example, the 10.7-unit L* range within P4 is larger than many between-package ΔE*ab values. Consequently, a production sorting system should operate at sheet or surface-zone level and should be validated on independent packages, rather than assigning a fixed color class to a region.

The current dataset can support descriptive matching of these eight packages. It cannot establish that the same color profile will recur in a new package from the same region, because stand, tree, log position, and processing batch were not replicated.

P5 (Katowice) was the most distinct package in the combined color profile, with low lightness and higher red and yellow coordinates. The chemical results below provide a plausible compositional explanation for this package-level pattern.

### 3.3. Chemical Composition Analysis of Oak Veneers

Across the eight packages, ethanol-soluble extractives ranged from 12.84% to 16.66%, cellulose from 36.10% to 41.10%, and lignin from 24.17% to 28.81% (Table 4). Extractives had the largest relative variability (CV ≈ 10%), while cellulose (CV = 4.5%) and lignin (CV = 5.9%) were more uniform. P5 (Katowice) had the highest extractive and lignin contents, whereas P6 (Krosno) had the lowest extractive content.

The chemical dataset contains one composite value per package and therefore does not quantify analytical or within-package chemical variability. Comparisons should be regarded as screening-level descriptors of the selected lots.

Package-level Pearson and Spearman analyses showed a strong negative association between extractive content and lightness (r = −0.80, *p* = 0.017; ρ = −0.75, *p* = 0.033; Table 5). Leave-one-package-out Pearson recalculations retained a strong negative coefficient in every case (r = −0.96 to −0.72), indicating that this result was not dependent on any single package. P3–P5 combined higher extractive contents with lower L* values, whereas P1, P6, and P8 were lighter and had lower extractive contents. This is consistent with the visible-light absorption of phenolic and tannin-rich extractives [3,12,13,33].

Lignin showed (Table 5) positive Pearson coefficients with a* (r = 0.67, *p* = 0.066) and b* (r = 0.54, *p* = 0.163), but the corresponding Spearman correlations were weak and non-significant (ρ = 0.13, *p* = 0.756 for a*; ρ = 0.19, *p* = 0.651 for b*). Leave-one-package-out recalculation showed marked instability: coefficients ranged from −0.18 to 0.77 for lignin/a*, from −0.15 to 0.75 for lignin/b*, and from −0.40 to 0.93 for lignin/L*. When P5 was excluded, the Pearson coefficients changed sign for all three color coordinates. Therefore, the apparent Pearson lignin/color associations are driven largely by P5 and do not support a general mechanistic interpretation across the dataset.

P5 (Katowice) had the highest lignin (28.81%) and extractive (16.66%) contents, the lowest mean L* (59.4), and the highest mean a* (8.7) and b* (20.6). Its lignin content was the only value outside the 24.17–25.50% range formed by the other seven packages, giving this observation disproportionate leverage in Pearson analysis. P5 should therefore be treated as an informative package-level case rather than evidence of a general lignin/color or regional relationship.

Cellulose content was not associated conclusively with any color coordinate (all *p* > 0.23). This agrees with the absence of strong visible chromophores in cellulose, while also emphasizing that the present chemical analyses do not resolve individual extractive compounds or lignin structural units [35,36].

The corrected analysis identifies extractive content as the only chemical descriptor significantly associated with a color coordinate at the package level. The previously stated stronger coefficients and unreported principal component analysis have been removed because they could not be reproduced from the tabulated dataset.

The compositional results therefore support a mechanistic interpretation of package color, especially for lightness, but they do not establish causality. Tree age, heartwood position, anatomical figure, and processing may covary with chemical composition and were not independently controlled.

For industrial quality control, chemical measurements may be useful for calibrating or validating optical classification models, although routine wet chemistry is too slow for sheet-by-sheet sorting. Future work should link rapid spectroscopy or machine vision to independently replicated chemical reference data.

In summary, the package-level results indicate that extractives are the most informative and robust measured chemical correlate of lightness, whereas the apparent lignin/color relationships were not robust to rank analysis or exclusion of P5. These conclusions are narrower than the original origin-based claims and reflect the available replication.

### 3.4. XRD Analysis and Reference to Native Cellulose

**Figure 2:**
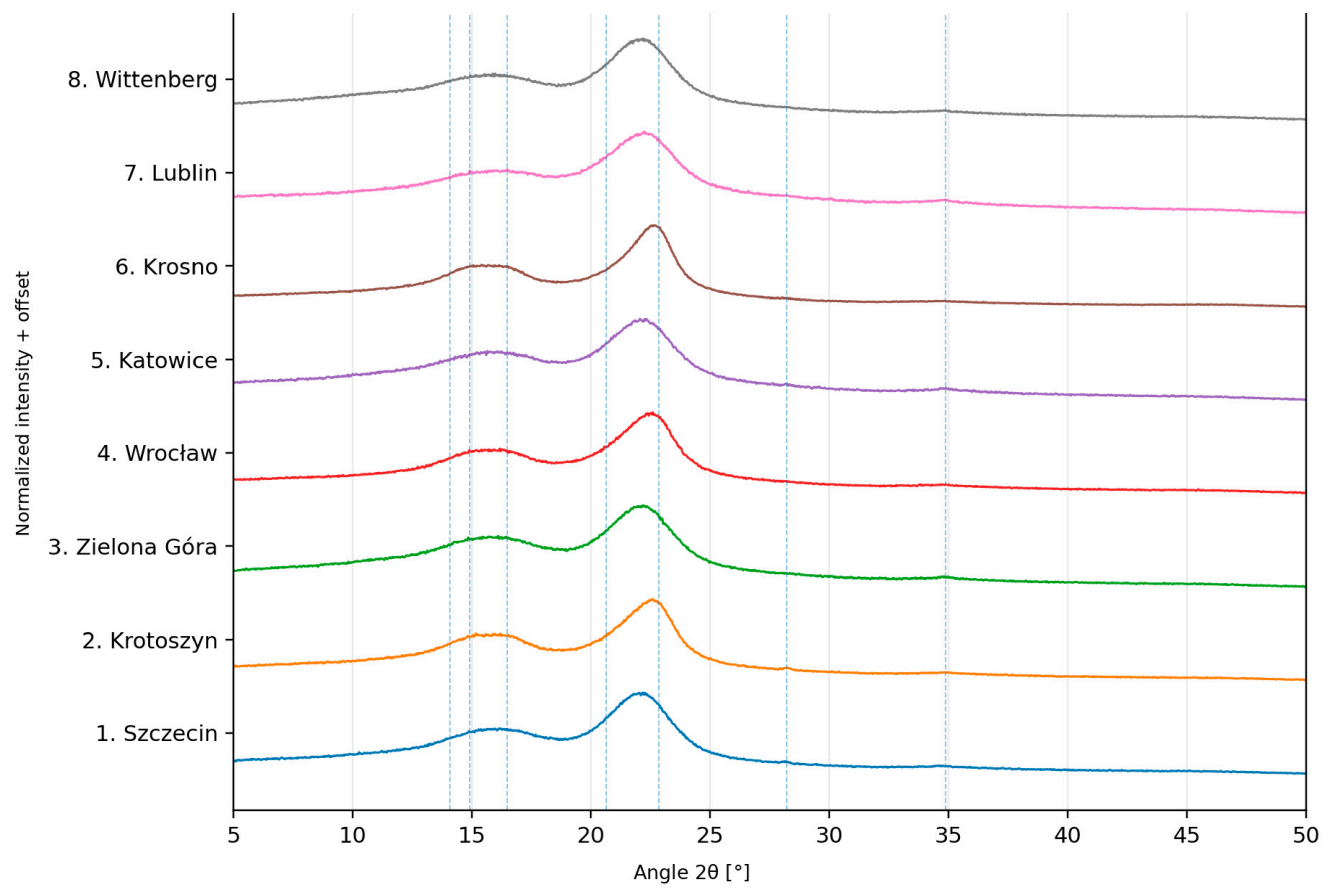
Normalized XRD diffractograms of P1–P8 (Szczecin, Krotoszyn, Zielona Góra, Wrocław, Katowice, Krosno, Lublin, and Wittenberg, respectively). Dashed lines indicate selected cellulose I reference positions.

**Figure 3:**
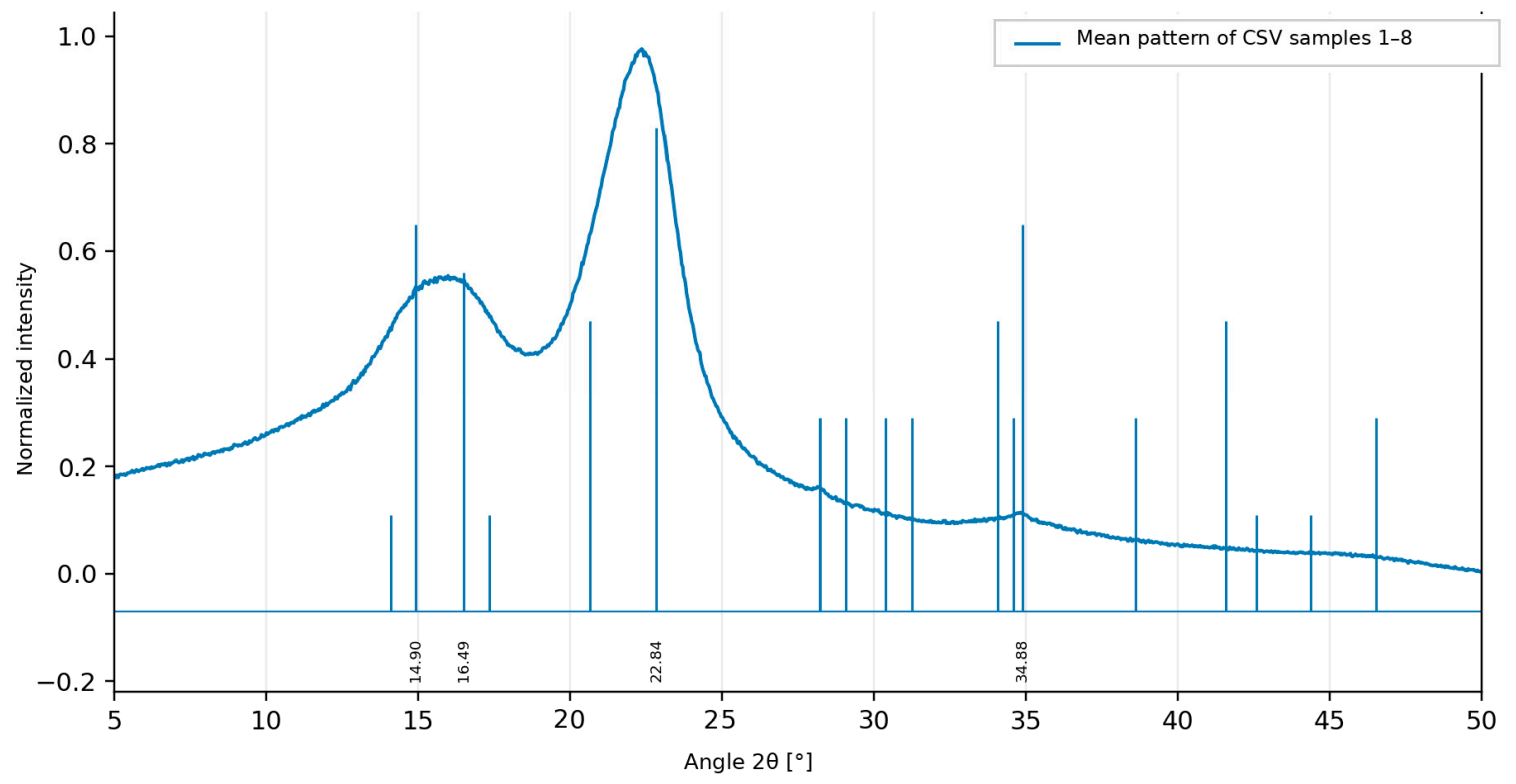
Mean normalized diffractogram of P1–P8 compared with selected reflections from the Native Cellulose 00-003-0289 reference card.

All eight diffractograms showed a broad low-angle feature near 15–17° 2θ and a dominant maximum near 22–23° 2θ (Figure 2), consistent with cellulose I. The principal package maxima ranged from 22.06° to 22.63° 2θ (Table 6). The reference comparison in Figure 3 and Table 7 is qualitative because the reference card is low precision and wood contains substantial amorphous hemicellulose and lignin.

Approximate CrI values ranged from 47.97% in P5 (Katowice) to 66.16% in P6 (Krosno), with a mean of 54.7%. These values are comparative indices, not absolute crystalline mass fractions, because the Segal method does not separate overlapping crystalline and amorphous contributions and the measurements were not corrected for preferred orientation [38,39].

The cellulose repeat unit is C6H10O5 and the polymer is written (C6H10O5)n; the earlier glucose formula C6H12O6 was incorrect. Agreement of the principal sample maximum with the reference reflection near 22.84° 2θ supports the presence of native cellulose I, while differences in peak intensity may also reflect specimen orientation and packing.

P6 (Krosno) had the highest I(002) intensity and approximate CrI, whereas P5 (Katowice) had the lowest. P2 (Krotoszyn) and P4 (Wrocław) also showed CrI values above the dataset mean. These observations characterize the tested specimens only and should not be generalized to their regions.

The full 5–110° scans contained 10,498 points per package. Detailed measurement conditions, complete scan verification information, the full reference reflection list, and a combined presentation of individual diffractograms are provided in the Supplementary Materials (Tables S1–S4 and Figure S1).

The main text retains the normalized comparison because it most clearly shows the common cellulose I pattern without duplicating the same information in multiple figures and tables.

### 3.5. Exploratory Associations Between XRD Descriptors and CIELAB Color

At the package level, CrI showed a positive but non-significant association with L* (r = 0.57, *p* = 0.139) and negative associations with a* (r = −0.56, *p* = 0.150) and b* (r = −0.63, *p* = 0.094) (Table 8). The position of the principal maximum was not significantly related to L* or a*, and its association with b* was also inconclusive (r = −0.68, *p* = 0.062).

I(002) peak intensity was positively associated with L* (r = 0.73, *p* = 0.039), but raw intensity is sensitive to specimen thickness, packing, and preferred orientation. Given n = 8, multiple exploratory tests, and one XRD specimen per package, this result should not be interpreted as evidence that cellulose ordering causes lighter wood. It is a hypothesis for replicated testing with standardized specimen preparation and independent packages.

### 3.6. Study Limitations and Implications for Industrial Sorting

The principal limitation is experimental unit replication: one package represented each regional label. Region is therefore aliased with stand, tree, log position, harvest, and processing batch. This study cannot estimate a regional variance component or validate a geographic classifier. A future design should include multiple independent stands, trees, logs, and production packages per region, with hierarchical mixed-effects analysis.

A second limitation is measurement reproducibility. The archived color files did not retain illuminant, observer, geometry, and black-calibration settings; chemical analyses lacked analytical replicates; and XRD was represented by one specimen per package with an empirical CrI calculation. These constraints require cautious, exploratory interpretation.

For manufacturing, the data indicate that sheet-level or zone-level color measurement is more defensible than assigning a fixed color to a regional label. With a larger replicated dataset, calibrated machine vision or artificial intelligence models could integrate CIELAB values, grain pattern, and chemical or spectroscopic proxies to support automated veneer matching. Such models would require external validation on new production batches.

## 4. Conclusions

This study explored whether combined colorimetric, chemical, and XRD descriptors can support quality control of eight commercial European oak veneer packages. It did not provide a replicated test of geographic origin because each regional label was represented by one package.

The largest difference among package means occurred in lightness, but within-package L* ranges reached 10.7 units and overlapped many between-package differences. Accordingly, the results support matching of the studied lots but do not support general origin-based sorting rules.

Among the measured chemical descriptors, ethanol-soluble extractives showed the clearest and most robust relationship with color: higher extractive content was associated with lower L* (Pearson r = −0.80, *p* = 0.017; Spearman ρ = −0.75, *p* = 0.033), and leave-one-package-out coefficients remained between −0.96 and −0.72. Apparent Pearson lignin trends were not reproduced by Spearman rank analysis and changed sign after exclusion of P5, so they cannot be interpreted as a general dataset-wide relationship.

All XRD patterns contained cellulose I features, and approximate Segal CrI values ranged from 48.0% to 66.2%. CrI/color associations were not statistically conclusive, while the positive intensity/L* association requires replication because diffraction intensity is sensitive to specimen preparation and orientation.

Combined optical and chemical characterization is therefore promising for package-level quality control, provided that future studies use independent replication, complete colorimetric metadata, analytical replicates, standardized XRD preparation, and external validation. These steps are necessary before geographic classification or automated AI-assisted veneer sorting can be achieved.

## Figures and Tables

**Table 1:** Characteristics and commercial provenance labels of the oak veneer packages.

ID	Regional Label (Country)	Producer Package ID	N (Sheets)	L (m)	B (cm)	P (m^2^)
P1	Szczecin, Poland	578428-12	32	2.30	21	15.46
P2	Krotoszyn, Poland	578052-13	33	2.30	10	7.59
P3	Zielona Góra, Poland	1656232-12	24	2.10	16	8.00
P4	Wrocław, Poland	565642-21	24	2.45	13	7.64
P5	Katowice, Poland	544954-14	32	2.25	13	9.36
P6	Krosno, Poland	576416-11	32	2.25	15	10.80
P7	Lublin, Poland	549219-14	32	2.50	10	8.00
P8	Wittenberg, Germany	576613-32	24	2.25	13	7.02

N = number of veneer sheets in the package; L = sheet length; B = sheet width; P = total veneer area. The producer package ID is an internal production batch traceability code. Dominant grain patterns were straight-grained in P1–P3, cathedral/arched in P4–P5, and linear-banded in P6–P8.

**Table 2:** Descriptive CIELAB statistics for ten measurement points in each veneer package.

Package/Region	Chromatic Variables	Average	Med.	Min	Max	Standard Deviation
P1 Szczecin	L*	69.7	69.1	67.4	73.5	1.91
	a*	6.8	6.9	6.2	7.4	0.37
	b*	19.8	19.9	18.8	20.6	0.59
P2 Krotoszyn	L*	66.8	67.1	64.0	68.6	1.29
	a*	7.1	7.3	6.6	7.7	0.35
	b*	19.4	19.6	18.2	20.5	0.72
P3 Zielona Góra	L*	62.0	62.8	56.6	65.7	2.58
	a*	7.2	7.3	6.6	8.3	0.51
	b*	20.1	20.2	16.5	21.0	0.55
P4 Wrocław	L*	62.0	60.9	57.8	68.5	3.91
	a*	6.9	6.8	6.2	8.1	0.64
	b*	18.1	18.1	16.5	19.7	0.78
P5 Katowice	L*	59.4	59.4	57.2	61.0	1.25
	a*	8.7	8.8	8.2	9.0	0.29
	b*	20.6	20.5	20.0	21.3	0.37
P6 Krosno	L*	68.0	68.3	64.1	72.9	2.89
	a*	7.2	7.4	5.8	8.6	1.03
	b*	18.8	18.7	17.8	20.4	0.81
P7 Lublin	L*	60.8	60.9	56.9	66.5	2.39
	a*	7.8	8.0	7.2	8.3	0.36
	b*	19.5	19.7	18.2	20.2	0.59
P8 Wittenberg	L*	68.6	68.4	68.0	69.8	0.59
	a*	8.1	8.2	7.6	8.5	0.25
	b*	18.7	18.7	17.8	19.3	0.38

**Table 3:** Pairwise ΔE*ab values calculated from the displayed package mean CIELAB coordinates.

	P1	P2	P3	P4	P5	P6	P7	P8
**P1**	0.00	2.94	7.72	7.89	10.50	2.01	8.96	2.03
**P2**		0.00	4.85	4.98	7.67	1.35	6.04	2.17
**P3**			0.00	2.02	3.04	6.14	1.47	6.81
**P4**				0.00	4.03	6.05	2.05	6.73
**P5**					0.00	8.91	1.99	9.41
**P6**						0.00	7.26	1.09
**P7**							0.00	7.85
**P8**								0.00

Interpretation used for descriptive comparison: ΔE*ab < 1, negligible; 1–2, very small; 2–3.5, noticeable; 3.5–5, distinct; >5, large. These thresholds do not account for within-package variance.

**Table 4:** Package-level chemical composition (% of oven dry mass).

Sample	Extractives * (%)	Cellulose (%)	Lignin (%)
P1	13.27	37.39	25.50
P2	13.69	41.10	24.90
P3	16.06	38.20	24.17
P4	15.91	40.90	24.55
P5	16.66	38.46	28.81
P6	12.84	40.26	25.31
P7	13.90	36.10	24.42
P8	13.90	38.16	25.05
Mean	14.53	38.82	25.34
Median	13.90	38.33	24.98
Minimum	12.84	36.10	24.17
Maximum	16.66	41.10	28.81
Standard deviation	1.45	1.77	1.47

* Oak extractives may include hydrolysable tannins (especially ellagitannins), non-volatile phenols, volatile compounds, and lipophilic substances [32].

**Table 5:** Exploratory package-level Pearson (r) and Spearman rank (ρ) correlations between chemical composition and mean CIELAB coordinates (n = 8; two-sided *p*-values). Each cell reports r (p)/ρ (p).

Component	L*	a*	b*
Extractives	r = −0.80; *p* = 0.017 ρ = −0.75; *p* = 0.033	r = 0.36; *p* = 0.380 ρ = 0.47; *p* = 0.240	r = 0.33; *p* = 0.426 ρ = 0.37; *p* = 0.365
Cellulose	r = 0.17; *p* = 0.691 ρ = −0.08; *p* = 0.844	r = −0.37; *p* = 0.366 ρ = −0.24; *p* = 0.568	r = −0.48; *p* = 0.232 ρ = −0.33; *p* = 0.420
Lignin	r = −0.26; *p* = 0.531 ρ = 0.26; *p* = 0.528	r = 0.67; *p* = 0.066 ρ = 0.13; *p* = 0.756	r = 0.54; *p* = 0.163 ρ = 0.19; *p* = 0.651

**Table 6:** Package-level XRD descriptors and approximate Segal crystallinity indices.

CSV/No.	Regional Origin	2θ Maximum [°]	Imax [Counts]	I(002) [Counts]	Iamorf. [Counts]	CrI [%]	Main Maxima 2θ [°]
P1	Szczecin	22.16	16,382.7	16,382.7	7540.3	53.97	22.24; 16.19; 28.13; 34.57
P2	Krotoszyn	22.60	14,718.0	14,718.0	6093.0	58.60	22.56; 15.22; 15.96; 28.19
P3	Zielona Góra	22.16	13,195.2	13,195.2	6565.3	50.24	22.25; 15.77; 34.80
P4	Wrocław	22.60	14,502.5	14,502.5	6156.3	57.55	22.57; 16.24; 34.72
P5	Katowice	22.06	11,958.1	11,958.1	6222.0	47.97	22.33; 15.75; 28.17; 34.72
P6	Krosno	22.63	20,053.9	20,053.9	6785.6	66.16	22.67; 15.46; 34.51
P7	Lublin	22.26	12,089.5	12,089.5	6100.2	49.54	22.21; 16.48; 6.58; 34.88
P8	Wittenberg	22.16	14,353.9	14,353.9	6738.9	53.05	22.14; 15.93; 34.85

**Table 7:** Selected reference reflections used for qualitative cellulose I identification.

No.	hkl	d (Å)	2θ (°)	I (%)
1	−1 0 1	6.28	14.091	20
2	unassigned	5.94	14.902	80
3	−1 1 1	5.37	16.494	70
4	1 0 1	5.11	17.340	20
5	0 2 1	4.30	20.639	60
6	0 0 2	3.89	22.842	100
7	1 3 0	3.16	28.218	40
8	0 4 0	2.57	34.882	80

**Table 8:** Exploratory package-level Pearson correlations between XRD descriptors and mean CIELAB coordinates (n = 8; two-sided *p*-values).

XRD Descriptor	L*	a*	b*
CrI	0.57 (*p* = 0.139)	−0.56 (*p* = 0.150)	−0.63 (*p* = 0.094)
2θ maximum	0.22 (*p* = 0.602)	−0.57 (*p* = 0.136)	−0.68 (*p* = 0.062)
I(002) intensity	0.73 (*p* = 0.039)	−0.53 (*p* = 0.172)	−0.44 (*p* = 0.269)

L* represents lightness, or how bright or dark a color is; a* represents the red-green axis; b* represents the blue-yellow.

## Data Availability

The original contributions presented in this study are included in the article/Supplementary Material. Further inquiries can be directed to the corresponding author.

## Supplementary Materials

The following supporting information can be downloaded at https://www.mdpi.com/article/10.3390/molecules31152712/s1: Table S1: Detailed XRD measurement conditions; Table S2: Identification data for the Native Cellulose 00-003-0289 reference card; Table S3: Verification of full scan reading; Table S4: Full reference reflection list; Figure S1: Combined individual XRD diffractograms of P1–P8.

## References

[1] Walinder M. (2006). The new journal Wood Material Science and Engineering. Wood Mater. Sci. Eng..

[2] Moya R., Berrocal A. (2010). Wood color variation in sapwood and heartwood of young trees of Tectona grandis and its relationship with plantation characteristics, site, and decay resistance. Ann. For. Sci..

[3] Gierlinger N., Jacques D., Gradner M., Wimmer R., Schwanninger M., Rozenberg P., Pâques L.E. (2004). Color of larch heartwood and relationships to extractives and brown-rot decay resistance. Trees.

[4] Kreber B. (1994). Understanding Wood Discoloration Helps Maximize Wood Profits.

[5] Nemeth R., Ott A., Takats P., Bak M. (2013). The effect of moisture content and drying temperature on the color of two poplars and Robinia wood. BioResources.

[6] Wiedenbeck J., Wiemann M., Alderman D., Baumgras J., Luppold W. (2003). Defining Hardwood Veneer Log Quality Attributes.

[7] Brunner C.C., Shaw G.B., Butler D.A., Funck J.W. (1990). Using color in machine vision systems for wood processing. Wood Fiber Sci..

[8] Ozarska B. (2003). A manual for decorative wood veneering technology. Project No. PN01.1600, Prepared for the Forest and Wood Products Research and Development Corporation and Funded by the Australian Government.

[9] Lutz J.F. (1977). Wood Veneer: Log Selection, Cutting, and Drying.

[10] Venet J., Keller R. (1986). Identification et Classement des Bois Francais.

[11] Hon D.N.S., Shiraishi N. (1991). Wood and Cellulose Chemistry.

[12] Klumpers J., Janin G., Becker M., Lévy G. (1993). The influences of age, extractive content and soil water on wood color in oak: The possible genetic determination of wood color. Ann. For. Sci..

[13] Mosedale J.R., Charrier B., Janin G. (1996). Genetic control of wood color, density and heartwood ellagitannin concentration in European oak (*Quercus petraea* and *Q. Robur*). Forestry.

[14] Janin G. (1987). Mesure de la couleur du bois; Intérêt forestier et industriel. Ann. For. Sci..

[15] Zanetti M., Mothe F., Merlin A., Janin G., LeMoguedec G., Goncalez J. (2003). Conséquences du vieillissement du bois de chêne sessile (*Quercus petraea* (Matt.) Liebl.) sur sa perception esthétique par les utilisateurs. Ann. For. Sci..

[16] Hoffmann P. (1987). Mesure de la Variabilite de la Couleur du Bois: Méthodologie et Influence de L’anatomie du Plan Ligneux et de la Variation Angulaire du Debit.

[17] Mazet J.F., Triboulot M.C., Janin G., Merlin A., Deglise X. (1993). Modifications de la couleur du bois de chenes europeens expose a la lumiere solaire. Ann. For. Sci..

[18] Sandoval-Torres S., Jomaa W., Marc F., Puiggali J.R. (2010). Color alteration and chemistry changes in oak wood (Quercus pedunculata (Ehrh)) during plain vacuum drying. Wood Sci. Technol..

[19] Resch H., Hansmann C., Pokorny M. (2000). The color of wood from white oak. Forsch.-Entwickl..

[20] Ferrari S., Allegretti O., Cuccui I., Morretti N., Marra M., Todaro L. (2013). A revaluation of Turkey oak wood (*Quercus cerris* (L.)) through combined steaming and thermo-vacuum treatments. BioResources.

[21] Todaro L., Dichicco P., Morretti N., D’Auria M. (2013). Effect of combined steam and heat treatments on extractives and lignin in sapwood and heartwood of Turkey oak (*Quercus cerris* (L.)) Wood. BioResources.

[22] Mitisor A., Istrate V. (1982). Technology of Veneers, Plywood and Fibreboards (In Romanian: Tehnologia Furnirelor, Placajelor si Placilor din Fibre de Lemn).

[23] Morosanu C. (2011). Study Regarding the Quality of Wood from European Oak and Sessile Oak Having as Destination Aesthetic Veneers. Ph.D. Dissertation.

[24] Dumitrascu E.A., Ciobanu V.D., Lepadatescu B. (2013). Valorization of wood resources for the cutting of decorative veneer in the context of sustainable development of Romanian forests. BioResources.

[25] McGavin R.L., Bailleres H., Lane F., Blackburn D., Vega M., Ozarska B. (2014). Veneer recovery analysis of plantation eucalypt species using spindleless lathe technology. BioResources.

[26] (2013). I.N.S. https://insse.ro/cms/ro/content/volumul-de-lemn-exploatat.

[27] Smolarek E., Kowalska J., Pałubicki B., Wieruszewski M. (2025). The Habitats of European Oak (Quercus) in Poland and General Oak Wood Color Issues. Forests.

[28] Scalbert A., Monties B., Janin G. (1987). Comparaison de Méthodes de Dosage des Tanins: Application à des Bois de Différentes Espèces.

[29] Stich K., Ebermann R. (1987). Oak peroxydase: Relationship with polyphenoloxydase. Holzforschung.

[30] (2012). Hardwood Round Timber—Qualitative Classification—Part 1: Oak and Beech.

[31] (1984). Paints and Varnishes—Colorimetry—Part 2: Color Measurement.

[32] Szynal D., Klimowicz A. (2026). The use of oaks in cosmetology. Pomeranian J. Life Sci..

[33] Gierlinger Notburga N., Wimmer Rupert R. (2004). Radial distribution of heartwood extractives and lignin in mature European larch. Wood Fiber Sci..

[34] Tolvaj L., Persze L., Lang E. (2013). Correlation between hue angle and lightness of wood species grown in Hungary. Wood Res..

[35] Rowell R.M. (2012). Handbook of Wood Chemistry and Wood Composites.

[36] Fengel Dietrich D., Wegener Gerd G. (1989). Wood: Chemistry, Ultrastructure, Reactions.

[37] Schwanninger Manfred M., Hinterstoisser B., Gierlinger N., Wimmer R. (2004). Application of Fourier Transform Near Infrared Spectroscopy (FT-NIR) to thermally modified wood. Eur. J. Wood Wood Prod..

[38] Segal L., Creely J.J., Martin A.E., Conrad C.M. (1959). An empirical method for estimating the degree of crystallinity of native cellulose using the X-ray diffractometer. Text. Res. J..

[39] Park S., Baker J.O., Himmel M.E., Parilla P.A., Johnson D.K. (2010). Cellulose crystallinity index: Measurement techniques and their impact on interpreting cellulase performance. Biotechnol. Biofuels.

[40] TAPPI (2015). Acid-Insoluble Lignin in Wood and Pulp, Test Method T 222.

