# Supplementary material for: Color Variability of European Oak Veneer Packages: An Exploratory Colorimetric, Chemical, and X-Ray Diffraction Study
Source: Molecules. 2026 Aug 4;31(15):2712. doi: 10.3390/molecules31152712 (PMC13468641; doi:10.3390/molecules31152712)
Supplement: Supplementary file 1 [file molecules-31-02712-s001.zip › molecules-4459512-supplementary.pdf]

Supplementary Materials

Color Variability of European Oak Veneer Packages: An Exploratory Colorimetric, Chemical, and X-ray Diffraction Study

Edmund Smolarek, Kinga Wichłacz-Szentner, Jarosław Jakubowicz, Maciej Jarzębski, and Marek Wieruszewski

Table S1. Detailed XRD measurement conditions.

**Parameter**	**Value**
Diffractometer system	EMPYREAN
Configuration	Bracket Flat Sample Stage
Sample stage	Stage for flat samples/holders
Goniometer	Theta/Theta
Anode material	Cu
K-Alpha1 wavelength	1.5405980 Å
K-Alpha2 wavelength	1.5444260 Å
Ratio K-Alpha2/K-Alpha1	0.500
Divergence slit	Fixed, 0.76 mm
Monochromator used	NO
Generator voltage	45 kV
Tube current	40 mA
Scan axis	Gonio
Scan range	5.01°–110.01° 2θ
Scan step size	0.0334225°
Scan type	CONTINUOUS
Time per step	29.845 s

Table S2. Identification data for the Native Cellulose 00-003-0289 reference card.

**Native Cellulose 00-003-0289 card parameter**	**Value**
Reference code	00-003-0289
Compound name	Native cellulose
Empirical formula	$C_6H_{10}O_5$ (anhydroglucose repeat unit)
Chemical formula	$(C_6H_{10}O_5)_n$
Crystal system	Monoclinic
Unit-cell parameters	a = 8.3500 Å; b = 10.2800 Å; c = 7.9600 Å
Cell angles	α = 90.0000°; β = 102.0000°; γ = 90.0000°
Measured density	1.60 g/cm3
Volume of cell	668.34 × 10^6 pm3
Subfiles	Carbohydrates; Forensic; Organic; Polymer
Quality	Low precision (O)
Creation Date	01.09.1953
Primary reference	Andress., Z. Phys. Chem. (Leipzig), 136, 279 (1928)
Card comment	Reflections were obtained from a fiber diagram; the card has quality O due to mean Δ2θ = 0.058.

Note: The original card reports historical phase-identification metadata. The chemically correct cellulose repeat unit is $C_6H_{10}O_5$ and the polymer formula is $(C_6H_{10}O_5)_n$.

Table S3. Verification of full scan reading.

CSV	Regional origin	Angle-intensity points	First point	Last point
P1	Szczecin	10498	5.02°; 3783.3	109.99°; 557.2
P2	Krotoszyn	10498	5.02°; 3375.6	109.99°; 515.4
P3	Zielona Góra	10498	5.02°; 3706.9	109.99°; 490.0
P4	Wrocław	10498	5.02°; 3557.2	109.99°; 558.9
P5	Katowice	10498	5.02°; 3661.9	109.99°; 559.5
P6	Krosno	10498	5.02°; 3828.3	109.99°; 497.4
P7	Lublin	10498	5.02°; 3674.5	109.99°; 533.7
P8	Wittenberg	10498	5.02°; 3988.8	109.99°; 523.7

Table S4. Full list of reflections in the Native Cellulose 00-003-0289 reference card.

No.	h	k	l	d [Å]	2θ [°]	I [%]
1	-1	0	1	6.28	14.091	20.0
2				5.94	14.902	80.0
3	-1	1	1	5.37	16.494	70.0
4	1	0	1	5.11	17.340	20.0
5	0	2	1	4.30	20.639	60.0
6	0	0	2	3.89	22.842	100.0
7	1	3	0	3.16	28.218	40.0
8	-1	2	2	3.07	29.063	40.0
9				2.94	30.378	40.0
10	1	3	1	2.86	31.249	40.0
11	3	1	0	2.63	34.062	60.0
12	-2	3	1	2.59	34.605	40.0
13	0	4	0	2.57	34.882	80.0
14	-2	3	2	2.33	38.610	40.0
15	2	4	0	2.17	41.584	60.0
16	1	2	3	2.12	42.612	20.0
17	3	0	2	2.04	44.370	20.0
18	-4	1	2	1.95	46.535	40.0

Blank hkl entries reproduce reflections that were unassigned in the reference card.

Figure S1. Combined individual XRD diffractograms.

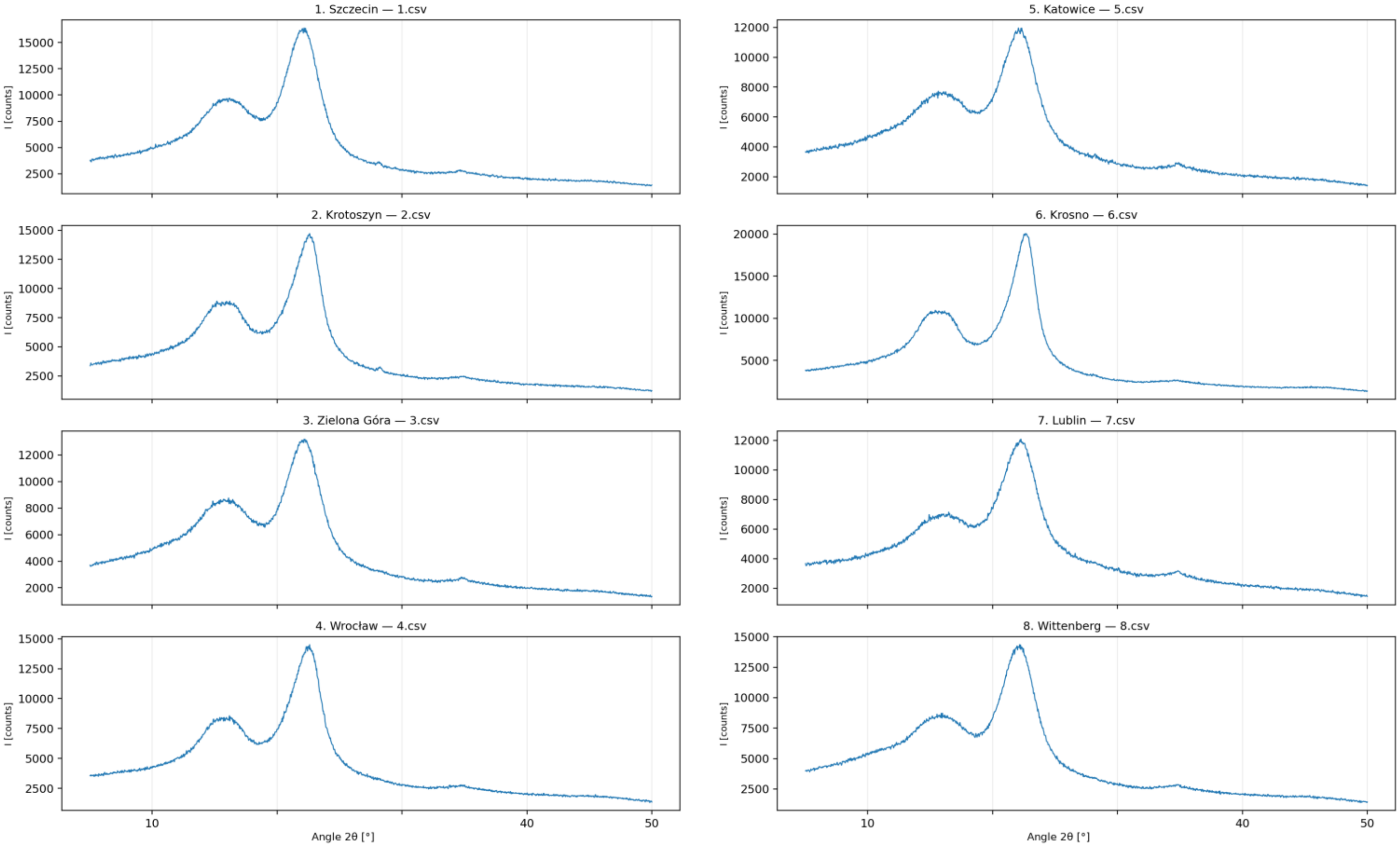

Figure S1. Individual XRD diffractograms of P1-P8. P1 = Szczecin; P2 = Krotoszyn; P3 = Zielona Góra; P4 = Wrocław; P5 = Katowice; P6 = Krosno; P7 = Lublin; P8 = Wittenberg.
